# Logic models for the evaluation of complex interventions in public health: lessons learnt from a staged development process

**DOI:** 10.1186/s12889-025-23171-8

**Published:** 2025-05-24

**Authors:** Stephan Voss, Julia Bauer, Michaela Coenen, Caroline Jung-Sievers, Graham Moore, Eva Rehfuess

**Affiliations:** 1 Chair of Public Health and Health Services Research, Institute for Medical Information Processing, Biometry, and Epidemiology (IBE), Faculty of Medicine, LMU Munich, Munich, Germany; 2 Pettenkofer School of Public Health, Munich, Germany; 3 Centre for Development, Evaluation, Complexity and Implementation in Public Health Improvement (DECIPHer), School of Social Sciences, Cardiff University, Cardiff, UK; 4 Wolfson Centre for Young People’s Mental Health, Cardiff University, Cardiff, UK

**Keywords:** Logic model, Theory of change, Complex intervention, Evaluation, Evidence-based public health, Municipal health promotion

## Abstract

**Background:**

Logic models are valuable tools when evaluating complex interventions in public health. They can improve the understanding of how an intervention works and interacts with its system, and facilitate clear communication regarding the intervention with stakeholders. There are different approaches to developing logic models. However, experiences with logic model development in practice, and the advantages or challenges associated with different approaches are rarely reported. This study describes and reflects on the staged development process of a logic model for the municipal public health intervention *Präventionskette Freiham* in Munich, Germany. Specifically, we aim to identify advantages and challenges associated with this process, and deduce lessons learnt for staged logic model development and logic model development in general.

**Methods:**

For the staged logic model development process, a first draft of the logic model was developed at the start of the evaluation. We then defined a priori milestones at which the model was to be revised. It was revisited and updated: (i) after the process evaluation, (ii) after a workshop with stakeholders, and (iii) at the end of preparations for long-term outcome evaluation. We discussed the advantages and challenges associated with the staged development process in a workshop within the research team, and obtained feedback on the usefulness of the logic model during a workshop with municipal stakeholders.

**Results:**

The logic model changed in multiple aspects during the different stages of development, mostly due to evidence obtained during the evaluation and as a result of feedback from discussions within the research team and with stakeholders. In the workshop with the research team, we found the staged process to be useful for facilitating reflection, for increasing plannability of logic model development, for integrating multiple perspectives and for increasing the validity of the logic model. Challenges were the need for rigorous documentation and flexibility in the process, as well as the management of resources. In the stakeholder workshop on the usefulness of the logic model, participants stated that the graphical visualization made it easier to handle complexity, and wished for the logic model to be re-used for future interventions.

**Conclusions:**

Developing a logic model in a staged process over the course of the evaluation of an intervention is a useful approach for stimulating reflection and obtaining a more realistic depiction of the intervention.

**Supplementary Information:**

The online version contains supplementary material available at 10.1186/s12889-025-23171-8.

## Background

Developing a logic model to conceptualize, plan, implement or evaluate interventions is a common recommendation in public health [1, 2]. According to the *Logic Model Developing Guide* by the Kellogg Foundation, a logic model is a graphical representation of the way an intervention is supposed to work, and of the intended or unintended outcomes it may produce [3]. Building on this, Rohwer et al. describe logic models as a “graphic description of a system”, emphasizing that a logic model should not only represent key elements of an intervention, but also the system in which that intervention is implemented [4].

Interventions in public health often consist of multiple components, include a variety of different actors and tend to have multiple causal pathways. Therefore, they can be considered complex interventions [5]. By visualizing key elements and mechanisms of an intervention, the system it is embedded in, and linking measures and outcomes, logic models represent a means of addressing and visualizing the complexity of public health interventions and of making implicit assumptions about how an intervention works explicit [6, 7]. Developing a logic model is particularly useful for conceptualizing an intervention in its early stage, and for specifying which outcomes to investigate when planning and conducting an evaluation [8]. Furthermore, visualizing an intervention has been suggested to be helpful for creating a shared understanding among the research team as well as stakeholders, and may thereby facilitate communication [2, 9].

In the following, we discuss logic model development mainly for the purpose of evaluating a public health intervention. Rehfuess et al. describe different approaches to how a logic model can be developed [10]. Logic models may be developed (i) a priori, at the start of the evaluation, with the initial draft not being revised at later stages; (ii) in an *iterative* process, in which the model can be modified at any time and as frequently as needed; or (iii) in a *staged* process, where the model is revised at predefined points in time (milestones) during the evaluation. Each approach has its strengths and limitations, and which is the most appropriate should be considered thoroughly by the research team. The a priori approach requires substantial resources to be dedicated to logic model development only at the beginning. However, it lacks flexibility, as it is unable to react to insights gained during the evaluation. This limitation can be avoided in approaches in which the logic model is revised during the evaluation [11]. In an iterative logic model development process, the initial logic model is merely considered as a draft that can be refined at any time. This can be a time-consuming process if done without restrictions, and the high degree of flexibility can come at the cost of transparency. Without rigorous documentation, it can be hard to track which change was introduced into the logic model, as well as when and why; with regards to the evaluation of an intervention, constant change may also lead to moving goalposts for the evaluation itself. A staged logic model development process seeks to strike a balance between the a priori and the iterative approach. As the model is only reworked at predefined time points, this approach grants flexibility while being more manageable and transparent than a fully iterative process.

Despite the broad recognition of the importance of logic models, their development process and their use for the evaluation of complex interventions in public health has been rarely described in detail [9, 12–15]. As a result, there is limited evidence on which advantages and challenges are associated with different approaches to developing a logic model. Furthermore, while improving communication with stakeholders represents a commonly stated advantage of logic models, their perceived usefulness in that regard is rarely reported in literature on real-world interventions. This study describes and reflects on the staged development of a logic model for the evaluation of *Präventionskette Freiham*, a municipal public health intervention in the city of Munich, Germany. We aimed to answer the following research questions: (1) What are the advantages and challenges associated with a staged logic model development process for public health interventions? (2) What can be learnt from this process, both for staged logic modelling and logic modelling in general? To do so, we first provide a detailed description of the development of the logic model for Präventionskette Freiham. The logic model was initially drafted and subsequently refined in several stages based upon insights obtained during the evaluation of the intervention and feedback from municipal stakeholders. Second, we discuss our lessons learnt for logic model development and use, both from the perspective of the research team and from the perspective of stakeholders.

## Methods

In this section, we describe the development process of a logic model for the municipal public health intervention *Präventionskette Freiham*. We decided to follow a staged approach as described by Rehfuess et al. [10], where the logic model is advanced over the course of the evaluation conducted by the research team. This approach would yield a logic model based on both consensus with stakeholders and empirical evidence, and potentially adaptable for the evaluation of complex interventions comparable to *Präventionskette Freiham*.

### Präventionskette Freiham

#### Intervention

*Präventionskette Freiham* is a municipal public health intervention that the administration of the city of Munich initiated in 2015. The intervention is the result of a cooperation between three municipal departments, the Department of Health, the Department of Social Services and the Department of Education and Sports. Freiham is a new residential development area that the city of Munich started to construct on its southwestern outskirts in 2016. The first residents moved to the district in late-2019. Upon completion, towards the end of the 2030s, at least 25,000 residents are expected to live in the district. Due to a large amount of social housing, many families with a low socioeconomic status are expected to live in Freiham [16]. Children from these households tend to grow up with disadvantages regarding health [17, 18] and education [19] compared to their peers. *Präventionskette Freiham* aims to promote equity regarding health, education and social participation for all children and adolescents living in Freiham by building an intersectoral network with key stakeholders.

*Präventionsketten* (literal translation: “prevention chains”) are a well-established concept in German-speaking countries, and exist in many cities [20]. One core assumption of these interventions is that transitions between different phases in life – e.g. from kindergarten to elementary school – can be a challenge for children and adolescents, especially for those from a disadvantaged socio-economic background [21, 22]. *Präventionsketten* aim to create a network between actors or institutions across different sectors and across different life phases to facilitate better access to and use of support services for children and their families. In doing so, the interventions are expected to improve the chances for young people to grow up without disadvantages regarding their health and education.

**Fig. 1:**
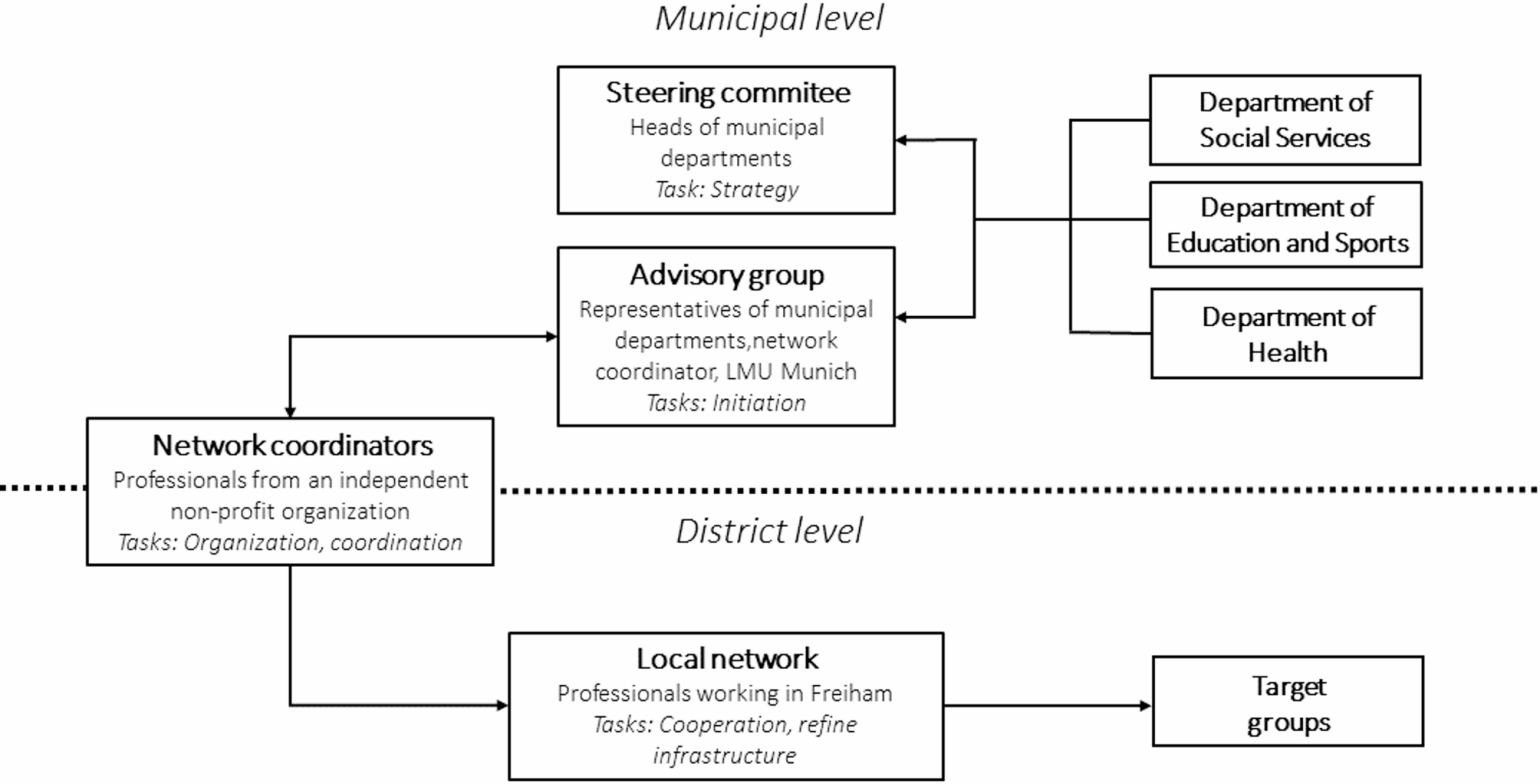
Core agents of Präventionskette Freiham

*Präventionskette Freiham* consists of several core agents: (1) The network coordinators, whose role it is to organize and guide a network of local professionals from different sectors in the Freiham district. (2) The local network of professionals working in institutions in Freiham, whose task it is to refine the support infrastructure for families according to the needs of this target group. Building this local network started in early 2020, shortly after the first residents moved to the Freiham district in late-2019. On the level of the municipal administration, there is (3) an advisory group which consists of representatives of the three departments, the network coordinators and one member of the research team at LMU Munich. The advisory group acts as the link between the municipal administration and the local network. Its task is to provide strategic impetus for collaboration for the local network and the municipal administration. (4) Furthermore, the steering committee consists of the heads of the three involved city departments and is responsible for long-term strategic decisions related to *Präventionskette Freiham*. A graphical overview of the core agents can be found in Fig. 1.

#### Evaluation

The evaluation of *Präventionskette Freiham* has been conducted by our research team at the Chair of Public Health and Health Services Research at LMU Munich. The evaluation process included three major phases. In phase 1, from 2019 to 2021, a process evaluation was conducted to obtain a deeper understanding of the implementation process and to identify facilitators and barriers for the implementation. To achieve this, we led expert interviews with the network coordinators and members of the local network in the Freiham district, and conducted a focus group with members of the advisory group of *Präventionskette Freiham* as key stakeholders of the intervention. The results of the process evaluation have been described elsewhere [23]. In phase 2, from 2022 to 2023, the main goal was to develop a strategy for outcome evaluation and, more specifically, implement a long-term monitor that included routine data on health, education and social indicators on children and adolescents in Freiham and other districts in Munich with a similar socio-economic structure. This monitor would allow us to compare these indicators over time with those in other districts without the intervention. To set up the monitor, an initial scoping review was conducted to extract relevant indicators [24]. After that, an eDelphi with experts on municipal health promotion was held to identify the most relevant indicators from the ones found in the scoping review [25]. As a next step, the research team concluded contracts with the Munich city administration and the physicians’ association in Bavaria to obtain the indicators identified in this eDelphi over the next years. During phase 2, the research team additionally conducted repeated online surveys with members of the local network of *Präventionskette Freiham* to gain insights on intermediate outcomes. The long-term monitor is designed to be a resource-effective way to track health, education and social development in the Freiham district for the next ten years (phase 3 of the evaluation), both for a long-term outcome evaluation and to provide feedback to stakeholders engaged with the *Präventionskette Freiham*.

### Logic model typology

There are different typologies for logic models. Rohwer et al. [4] distinguish between two types of logic models: *System-based logic models* place emphasis on the system in which the intervention takes place. In contrast, *process-oriented logic models* focus on the pathways leading from the intervention to its various outcomes. A different yet overlapping typology for logic models has been described by Mills et al. [26]. Here, four types of logic models are distinguished based on (i) whether logic models consider contextual factors or not and (ii) whether they seek to portray the relationships between logic model elements or not. In this typology, a *Type 1 Logic Model* lists all relevant factors of an intervention, excluding context. A *Type 2 Logic Model* includes this context. Congruently, a *Type 3 Logic Model* shows the relationships between different factors of an intervention without contextual factors, while a *Type 4 Logic Model* incorporates relationships with contextual factors. Type 4 Logic Models are the most comprehensive and, in theory, best suited for visualizing how complex interventions interact with the context into which they are introduced. However, due to their complexity, they are often hard to understand and therefore less suitable for achieving consensus among and/or communicating with a broad range of stakeholders. Which type of logic model should be used, depends on its purpose; in practice a mixture is often applied.

### Staged development of the logic model

#### Overview of the staged development process

For the staged logic model development process over the course of the evaluation, we as the research team defined a priori milestones, at which the logic model was to be revisited and revised based on specific inputs (Table 1). Each new version of the logic model should reflect the research team’s understanding of the intervention at that specific stage, with regards to key domains, context and mechanisms for change. We pursued two objectives with staged logic modelling: On the one hand, the logic model was supposed to guide the evaluation, particularly for designing interview guides and surveys, and to uncover potential evaluation blindspots. On the other hand, it was intended to be an output of the evaluation process. Integrating insights from data collected during the evaluation and feedback obtained from stakeholders would yield an enhanced logic model based upon both evidence and consensus; this evaluation output could be useful for planning, implementing and evaluating similar interventions – specifically other *Präventionsketten* – in the future.

**Fig. 2:**
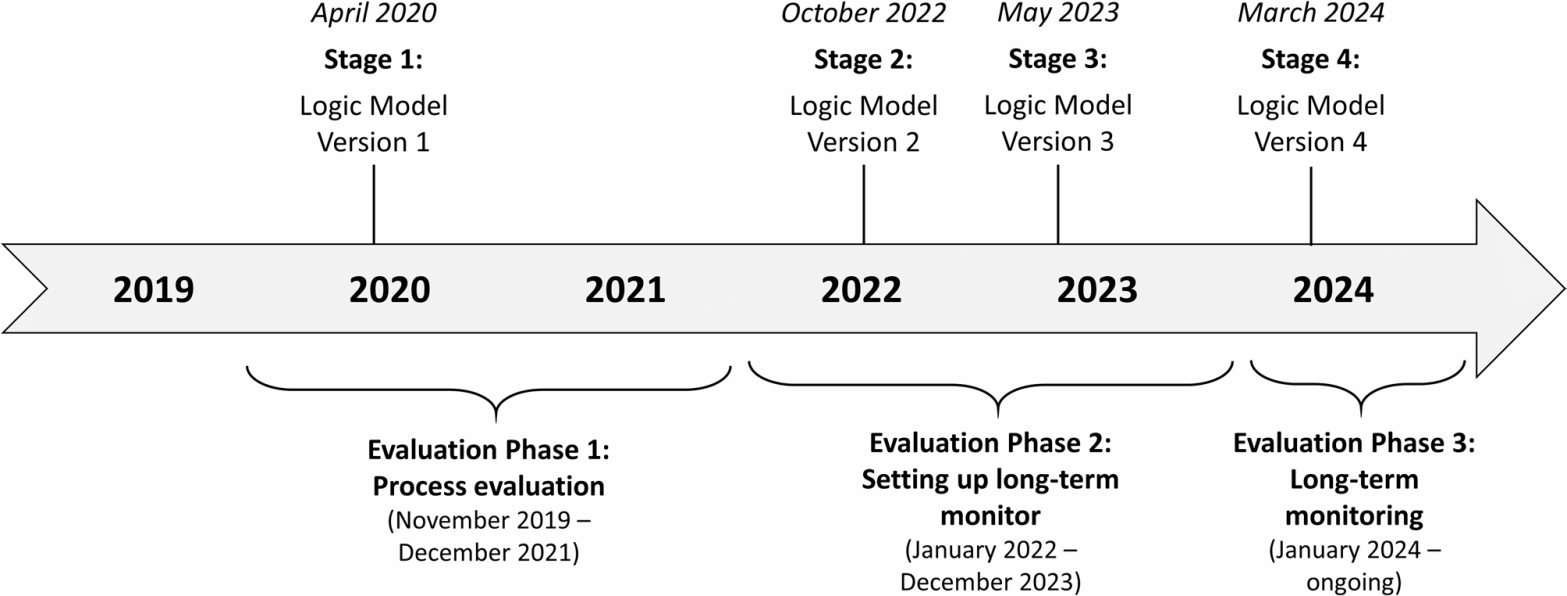
Timeline of the logic model development process during the evaluation of Präventionskette Freiham

Changes from one version to the next were based on insights gained upon reaching the specific milestone. All changes from one version to the next were documented by the first author in a separate document, and a rationale was provided for each change. We distinguished three types of changes: those affecting the *structure* of the logic model, those concerning the *content* of logic model domains and those affecting *wording* only. The timeline of the logic model development process is shown in Fig. 2. All logic model versions were initially developed in German and were later translated into English.

**Table 1:** Overview of the staged logic model development process for *Präventionskette Freiham*

Stage	Version	Milestone	Inputs
Stage 1	Logic model version 1	At the start of evaluation phase 1	• Research team reflections • Document review • Literature review
Stage 2	Logic model version 2	After the end of evaluation phase 1	• Research team reflections • Expert interviews with members of the local network and network coordinators • Focus group with members of advisory group
Stage 3	Logic model version 3	After workshop with advisory group	• Feedback from workshop with advisory group
Stage 4	Logic model version 4	After the end of evaluation phase 2	• Research team reflections • Document review • eDelphi on outcome indicators

#### Stage 1

The initial draft of the logic model was developed in April 2020. The initial logic model (version 1) was designed to create hypotheses for the evaluation of *Präventionskette Freiham* and to guide the evaluation. It was used to develop the guides for the expert interviews and the focus group during the process evaluation. Getting started, we made two important decisions. First, we decided to use a system-based template as described by Rohwer et al. [10], as emphasis should be placed on the system or setting where the intervention was implemented. The setting of *Präventionskette Freiham* as a new residential development area had hardly been addressed in public health research so far and thus merited specific attention. Second, we decided to develop a Type 2 logic model according to the typology by Mills [26] (i.e., presenting all relevant elements of the intervention, including context, but not depicting the relationships between these elements), as we wanted to avoid complexity to increase the logic model’s comprehensibility, especially for communication with various stakeholders. The first version of the logic model was based mainly on internal and official documents on the intervention, existing literature on comparable municipal public health interventions and iterative discussions within the research team.

#### Stage 2

Version 2 of the logic model was developed in October 2022, after the research team had completed data analysis of the expert interviews and the focus group as part of the process evaluation. This version was based on learnings from evaluation phase 1, and on reflections within the research team.

#### Stage 3

Version 3 of the logic model was developed in May 2023. During this stage, we incorporated feedback from a workshop with the advisory group of *Präventionskette Freiham*. This was the first stage where stakeholders outside the research team were engaged in the logic model development process. As some members of the advisory group had been involved in planning and implementing the intervention for years, they were considered to be key stakeholders with detailed insights on the intervention. The workshop was part of a regular meeting of the advisory group in March 2023 and took one hour. All data was collected anonymously at the workshop. Therefore, according to the General Data Protection Regulation (GDPR) of the European Union, no informed consent was required for participation. Additionally, we obtained a waiver from the Ethic Committee of the Medical Faculty at LMU Munich (22–0910) which confirmed that no ethics approval was required. However, we informed members of the advisory group in the beginning about the objectives and the procedure of the workshop. Based upon this information, they could decide whether they wanted to participate. One member of the research team (MC) was also part of the advisory group, but did not participate in the workshop. At the start, the current version of the logic model was introduced by the first author. Participants were then asked to reflect about each domain of the model, notably whether they would be able to identify aspects that needed to be changed. All statements were documented by the research team, maintaining the anonymity of participants. The feedback from the workshop was then integrated into the logic model.

#### Stage 4

Version 4 of the logic model was finalized in March 2024. We added insights gained during the development of the long-term monitor, mainly indicators considered relevant for the evaluation in the eDelphi in evaluation phase 2. Furthermore, we reviewed internal documents on *Präventionskette Freiham* that had not existed during our first document review in stage 1, and discussed our learnings regarding the intervention in several internal meetings. Insights from both sources were also incorporated into the model.

### Workshops to develop lessons learnt

#### Workshop within the research team

In September 2023, five members of the research team (CJS, ER, JB, MC, SV) participated in an internal workshop. The aim was to reflect on the advantages and challenges associated with the staged logic model development process to date. The workshop took place at the Chair of Public Health and Health Services Research at LMU Munich as a face-to-face meeting and took one and a half hour. Two members of the team took notes (ER, SV). The findings from this workshop were further developed in several additional short and smaller-group meetings.

#### Workshop with advisory group on usefulness of logic model

Logic model version 4 was presented to members of the advisory board in a workshop. The aim was to explore their perspectives on the usefulness of using a logic model and on the underlying development process. This workshop was conducted as part of a regular meeting of the advisory group in March 2024 and took one hour. As with the first workshop with the advisory group, participants did not have to provide informed consent according to the GDPR. Nevertheless, members of the advisory were informed at the start on the objectives and the procedure of the workshop, and could decide based upon this information whether they wanted to participate. The member of the research team that was also part of the advisory group (MC) did not participate as a member of the advisory group in the workshop, but recorded all statements anonymously in a protocol. The first author moderated the workshop. Version 4 of the logic model was introduced to participants before the following specific questions were asked:

- In which ways has the logic model expanded your understanding of the intervention?
- What are benefits of a logic model when implementing municipal interventions like *Präventionskette Freiham*?
- Which needs do you have regarding a logic model or the development of a logic model?

Workshop participants wrote down their answers to these questions, which were then collected by the moderator and a member of the research team and discussed in plenary. The answers obtained for each question were photographed for documentation.

## Results

### Logic model development

**Fig. 3:**
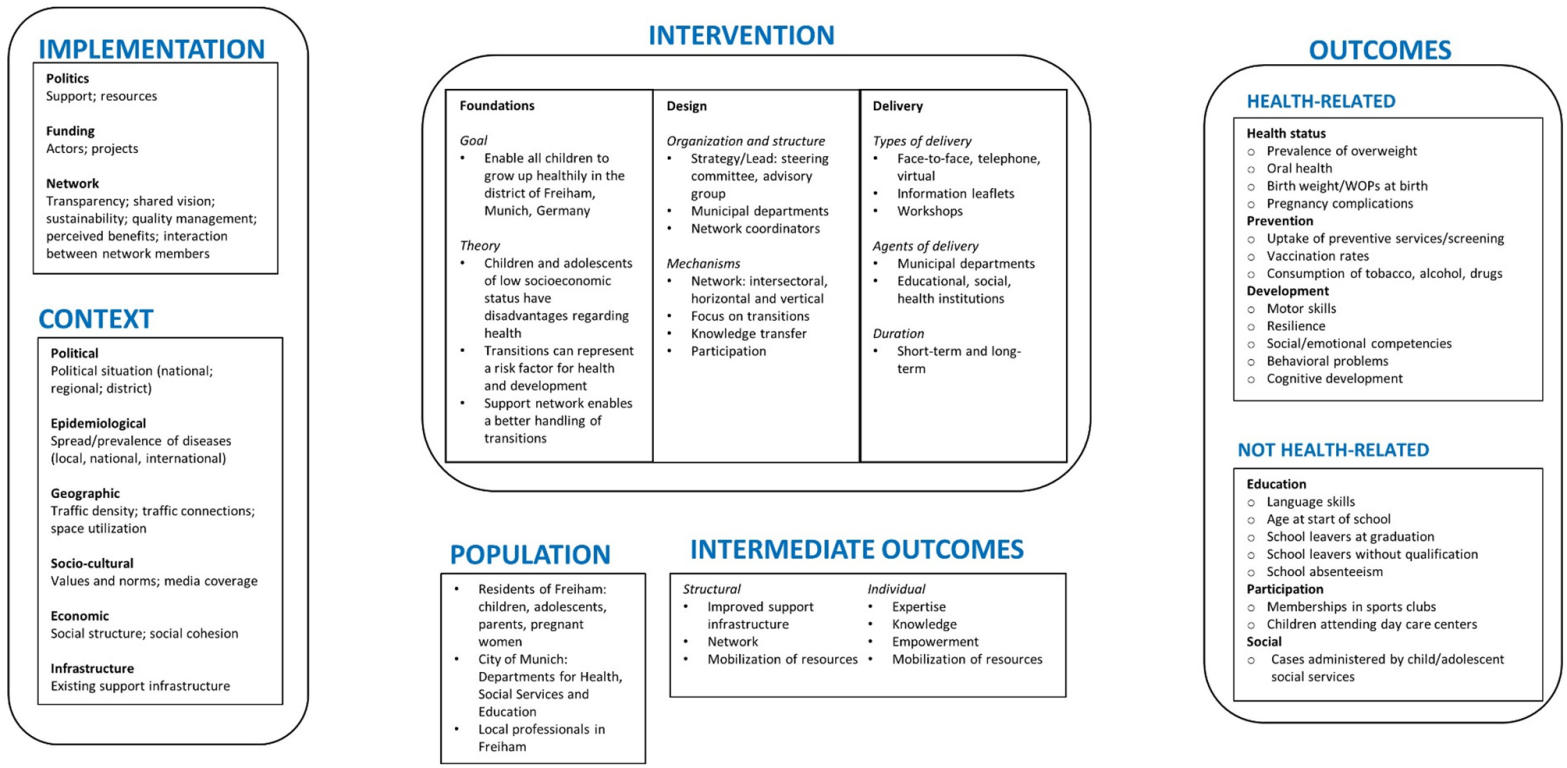
Version 1 of the logic model for Präventionskette Freiham

**Fig. 4:**
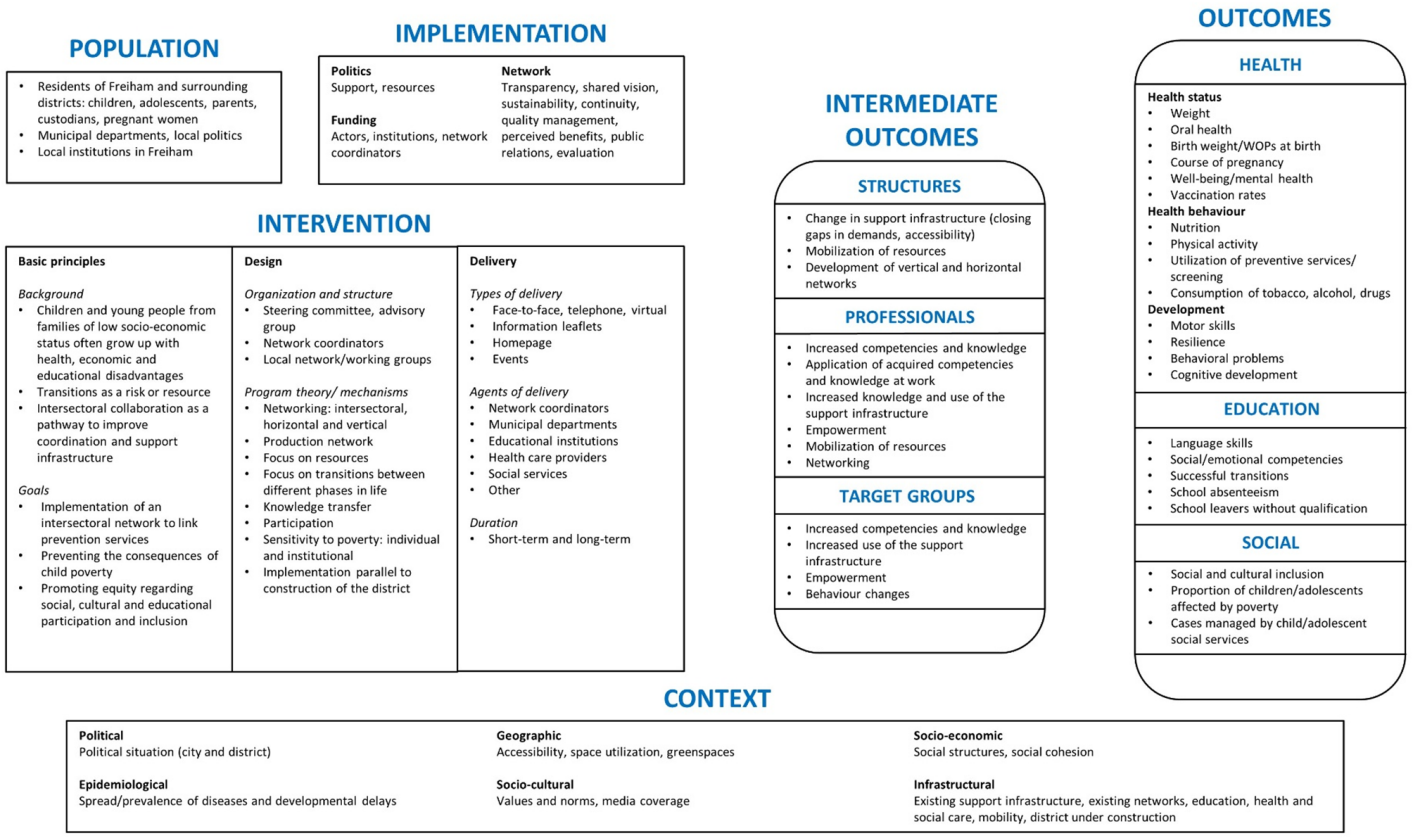
Version 4 of the logic model for Präventionskette Freiham

The initial logic model (version 1) is shown in Fig. 3, the final logic model (version 4) in Fig. 4. The intermediate versions (version 2 and 3) can be found in Supplementary Figs. 1 and 2, respectively. Stage-specific changes in the logic model are reported in Supplementary Table 1, with the exception of minor changes in wording. For each change, we documented the domain and sub-domain affected and the type of revision made, and provide a detailed description of the revision as well as the input and the rationale for the revision.

The first version of the logic model in stage 1 included six domains: *Population*, *Intervention*, *Implementation*, *Context*, *Intermediate Outcomes* and *Outcomes.* Content within *Population* and *Intervention* was mainly based on internal documents relating to *Präventionskette Freiham*. *Implementation*, *Context*, *Intermediate Outcomes* and *Outcomes* were based on previous research on *Präventionsketten* in Germany [27–31] and other municipal public health interventions with a focus on providing better services for children and families [32–34], as well as discussions within the research team. To develop sub-domains for the relevant contextual factors, we used the Context and Implementation of Complex Interventions (CICI) framework [35]. *Intermediate outcomes* comprised two sub-domains (structural vs. individual), whereas outcomes were differentiated as “health-related” and “non-health-related” outcomes.

In stage 2, changes to the logic model were based on insights gained during the process evaluation in evaluation phase 1. In addition to some minor changes in the domain *Intervention* that reflected our refined understanding of the intervention, major revisions to the logic model were made in the domain *Implementation*, as facilitators and barriers to the implementation were a main focus of the qualitative expert interviews with members of the local network and the network coordinators and the focus group conducted with members of the advisory group. In the interviews and the focus group, participants considered the tight funding situation of many institutions as an important barrier to a stronger engagement of local professionals in the network of the intervention. Other networks for families and children in the Freiham district that had been established before the implementation of *Präventionskette Freiham* were also stated to be an important factor by interviewees, and therefore added to the logic model as a context factor.

During stage 3, the logic model was revised following the workshop with the advisory group. Seven members of the advisory group participated in this workshop. All changes made to the logic model were based upon their feedback. Although participants emphasized different aspects in their feedback, all changes made found broad consensus. Workshop participants mainly stated that the current model placed too much emphasis on health compared to other sectors. In response, we revised the relevant part in the domain *Intervention* and divided *Non health-related outcomes* into two new sub-domains: *Educational* and *Social* outcomes. Furthermore, many smaller changes across all domains of the logic model were made to match the descriptions of the intervention with the understanding of municipal stakeholders. Based on discussions during this workshop, we also added several new aspects to program theory in the corresponding subdomain that had emerged as relevant during the implementation. In this context, we understand program theory as the mechanisms by which an intervention contributes to achieving its outcomes [36].

Changes made during stage 4 and resulting in logic model version 4 were mostly implemented as a result of our reflections on the current state of the logic model in research team discussions and due to insights gained in the eDelphi with experts to identify key indicators for long-term monitoring [25]. As eDelphi participants highlighted the importance of intermediate outcomes for measuring the effectiveness of the intervention, we placed more emphasis on this domain. The *Intermediate outcomes* were positioned more centrally within the model and divided into three sub-domains (*Structures*,* Professionals*,* Target groups*); the content of these sections was then expanded. Furthermore, as new wording on the background and goals of *Präventionskette Freiham* had emerged in official documents of the intervention, we revised these parts of the logic model to match the new wording.

### Advantages and challenges associated with the staged logic model development

In the workshop by our research team on learnings from the logic model development process and several subsequent short or smaller-group meetings, we identified several advantages that the staged approach offered compared to a priori or fully iterative approaches to logic model development:

- **Facilitate and focus reflection**: Each revision forced us to engage in a structured way with previous assumptions, and in which ways they had turned out to be valid or not. While this could also have been achieved with an iterative logic model development process, the staged process acted as guidance regarding which parts of the model the deliberation would focus on. After the process evaluation, it became clear that *Implementation* and *Context* would be the main domains to discuss, as these had been investigated in the expert interviews and the focus group. Furthermore, knowing that one of only a few revisions was imminent, made it easier to ensure that we would set aside time to reflect on the model.
- **Support planning for the research team**: While updating the logic model was a time-consuming process, doing it in a staged manner made it easier to plan ahead regarding what to do at which point in time, and with respect to the resources that would need to be allocated. Overall, this increased the predictability of the development process compared to a less structured iterative process.
- **Integrate variety of perspectives**: As complex interventions, public health interventions usually include actors with different backgrounds. Integrating the perspectives of these different actors can help to make the logic model more complete, and can enable each actor to reflect on blind spots and biases they may have due to their specific background. Our initial logic model placed much emphasis on health, both when describing the intervention and when listing outcomes. This was pointed out and criticized by stakeholders from educational and social sectors at the workshop with the advisory group. Consequently, this was corrected in the next version. While feedback from multiple stakeholders can also be included in other approaches to designing a logic model, a staged approach promotes this in a very explicit and transparent manner, as obtaining such feedback can, for example, be integrated as a stage of its own.
- **Increase validity**: The first version of the logic model was created mostly based on reflections within the research team while drawing on documents and a literature review. As a consequence, the accuracy of many statements in the model was unclear. Even when there was evidence from other interventions, it was uncertain to what extent such evidence would be transferable to the context of *Präventionskette Freiham*. When revising the logic model alongside the evaluation process, we were able to gradually substitute theory and indirect evidence with direct empirical insights from *Präventionskette Freiham*, and develop a more realistic model of the intervention. For example, the existence of other networks for families and children in the Freiham district had played no relevant role when planning *Präventionskette Freiham*, but it became clear during the process evaluation that this was a major factor influencing success or failure of the intervention. Consequently, these networks were added to the logic model. Indeed, most parts of the *Implementation* and *Context* domains of the logic model were validated by the research process, and thus became more trustworthy.
- **Increase utility and transferability**: Following this increased trustworthiness, the staged process developed a logic model that we felt more confident to use, both in team-internal meetings and in workshops with municipal stakeholders. At the time of the workshop in March 2024, the Munich city administration was planning a new *Präventionskette* in another new residential development area. As version 4 of the logic model for *Präventionskette Freiham* was based both on evidence and on stakeholder consensus, we decided that we could transfer this to the new intervention with minor adaptations, and therefore start the evaluation of the new *Präventionskette* on a solid theoretical foundation.

In addition to these benefits, we also identified some challenges associated with the staged development process:

- **Need for documentation**: Changes for each version of the logic model need to be documented accurately during a staged development process. What to record should be considered at the start of the process. Changes should be recorded in a structured manner, providing type of change (structure, content, wording) and a rationale for each decision, to make revisions traceable. This documentation increases the transparency of the logic model development process and can be useful for later discussions or questions regarding the revisions. However, the records should meet the right level of detail. Too much information can be overwhelming, too little detail obscure transparency.
- **Need for flexibility**: While a staged process requires predefined milestones, there may be good reasons for deviating from prespecified revision points. Initially, we had scheduled the workshop to obtain stakeholder feedback as an early step in our logic model development process. The workshop was initially planned for mid-2020, during stage 2. However, due to the COVID-19 pandemic and the contact restrictions imposed, we were forced to postpone the workshop. Likewise, at stages 2 and 4 we had intended to incorporate mainly insights from evaluation phase 1 and evaluation phase 2, respectively. However, when we started to work on these revisions it became clear in internal discussions that our understanding of the intervention had evolved from the prior version. Therefore, we decided that these changes should also be incorporated. While it is reasonable to predefine rules according to which the logic model should be revised, it is also reasonable to re-evaluate these rules and to assess whether, where and when to deviate from them in justified cases. Otherwise, one might risk overlooking relevant developments or insights. For example, while the stage 4 logic model was initially planned to be reworked based upon insights from evaluation phase 2, we realized during the process that we should also take other sources into account, particularly new documents that had been published since the first stage. Likewise, while stage 4 was initially planned to be the final version of the logic model, future iterations, informed by the long-term monitoring of *Präventionskette Freiham*, are likely to be relevant.
- **Managing resources**: Developing a logic model in a staged process represents a substantial time investment over a prolonged period, and this challenge is accentuated the more individuals are involved. Although revising the logic model and documenting the changes was done by the first author, requiring several days at each stage, each revision was also discussed by the research team. While the overall temporal expenditure for each team member was limited (one or several hours at each stage), the involvement of several people and repeatedly adding the logic model to the agenda of team meetings represented a considerable investment for the whole team. This was even more eminent at stage 3, where the logic model was also discussed with the advisory group.

### Feedback on the final logic model from municipal stakeholders

Nine members of the advisory group participated in the workshop to discuss version 4 of the logic model, with two joining virtually on Webex. General feedback on the logic model was that it was “very complex”, but that it provided a good overview of the intervention. Asked whether the logic model had expanded their understanding of the intervention, participants stated that the graphical overview reduced the complexity of the intervention to a simpler and condensed form, which was considered very helpful in providing an overview. Others pointed out that listing of contextual factors had been useful, as this identified gaps and needs that they would have to work on in the context of *Präventionskette Freiham*. Participants also highlighted that the distinction between outcomes and intermediate outcomes had been important for advancing their understanding of how the intervention worked. Based upon the logic model, it was discussed how specific outcomes could be measured and which departments of the city administration could provide the data.

Regarding benefits of the logic model, participants responded that it offered simplicity, and therefore could be used for communicating with internal and external partners. Furthermore, the logic model was considered a valuable tool for planning, analysis and quality control, and it was stated that it helped to keep the broader picture in mind.

Regarding their needs for the logic model or its development process, participants expressed that they wanted it to be transferable to other projects, especially to a new *Präventionskette* that the Munich city administration was planning in another residential development area at the time of the workshop. Others desired a digital version of the logic model that could be supplemented with data from the evaluation process or that could include references for the different domains. Furthermore, it was stated that a logic model should aim for a good balance between completeness and comprehensibility.

## Discussion

In this study, we developed a logic model for the municipal public health intervention *Präventionskette Freiham* in a staged process. The logic model was intended to guide the evaluation of this complex intervention, and was updated during the evaluation. While the overall structure of the logic model remained mostly unchanged during this process, the content of the different domains was revised significantly in several parts based on document reviews, data collected during the evaluation, feedback from stakeholders and reflections within the research team. Reflecting on the staged development process within the research team, the approach supported us in facilitating and focusing our reflections on the model, in planning the research process, in incorporating a variety of perspectives into the development process and in increasing the validity, utility and transferability of the logic model. Obtaining feedback on the usefulness of the version 4 logic model from stakeholders, they stated that the visual representation of the intervention – while complex – had made it easier to conceptualize and understand the intervention and to identify specific tasks to work on. Furthermore, they suggested that the logic model be adapted for similar future interventions.

Complex interventions are rarely put into practice exactly as planned, as they often change in a context-specific manner [37]. One criticism of logic models is that they tend to be too static and cannot depict the complexity of real-world implementation [38]. Developing a logic model in a staged process allows for changes to happen and for these changes to be documented; contrasting the different stages of the logic model with each other provides an overview of how the intervention evolved. According to the program theory of *Präventionskette Freiham*, managing transitions from life stage (e.g. kindergarten) to another (e.g. elementary school) is a core mechanism by which the intervention seeks to achieve its intended outcomes. However, during implementation, it became evident that other aspects, such as focusing on resources and making professionals aware of the consequences of poverty in children, played an equally important role. This topic was discussed in the workshop with the advisory group in stage 3 of logic model development, and the model was revised accordingly. Engaging practitioners from the advisory group with logic model development proved valuable for grounding the model in their real-world experience. Engaging these stakeholders even earlier might have led to improved data collection, specifically through informing the interview guides and the digital survey. While it is important to discuss a logic model early on with policy and practice stakeholders, as we had planned initially, we suggest that it is also advisable to involve stakeholders in reflections on the model at later stages of program implementation, when practical insights have already been gained.

Craig (2013) suggested that logic models should not be considered prescriptive, but that users should engage with them critically based on available evidence and context [8]. It can be considered a main advantage of a staged logic model development process that stages, where reflections must take place, are required and pre-specified. Consequently, this will likely yield a more realistic and more valid logic model of a given intervention. While this outcome could also be achieved with an iterative development process, the staged process helps to strike a balance between the need to adapt the model based on new insights and the need to limit time and resources spent on advancing the logic model.

Still, developing a logic model in a staged process is time-consuming and resource-intensive. Therefore, when deciding to pursue this route during the planning stages of the evaluation of a complex intervention, one needs to explore the potential benefits and whether this investment is worth the effort. In our research, being able to re-use the logic model for a similar future intervention was a desire expressed by municipal stakeholders. However, such applications may require changes to the logic model. One core assumption regarding complex public health interventions is that they are highly dependent on context [35]; therefore, with reference to realist evaluation, what works in one setting may lead to failure in another [39]. Transferring a complex intervention from one population, setting or context to another usually requires it to be adapted in several regards [40]. Sometimes the adaptation of a logic model may not be suitable at all. The ADAPT guidance for adapting interventions to new contexts suggests to carefully examine the existing intervention and the context, in which it was implemented, before deciding whether to adapt an intervention or not [41]. A more realistic and valid logic model of an intervention, appropriately developed in a staged development process, can provide valuable insights for such a decision, and potentially increase the likelihood of successful adaptations.

### Lessons learnt and implications for future research

Our research offers several lessons for developing logic models for public health interventions. Some of these also implicate future research on logic modelling.

Some lessons learnt specifically refer to the development of logic models in a staged manner: For one, complex interventions are often characterized by multiple, long causal pathways, that may interact with each other and that are often not clearly specified. A staged logic model development process can document how the understanding of these pathways evolves over the course of an evaluation. To fully reflect this evolution, another revision of the logic model may be necessary after the completion of the outcome evaluation of *Präventionskette Freiham*, as data collection and data analysis was not completed during stage 4 of logic model development. Furthermore, we argue that a staged development process may be helpful for obtaining a more easily transferable logic model. However, it is unclear whether and to which extent this proves to be feasible in practice. The logic model for *Präventionskette Freiham* will be adapted for the implementation of a *Präventionskette* in Neufreimann, another new residential development area in Munich. In this case, the logic model is intended to be used for program planning by the coordinators of the intervention. A recent study by Glasgow et al. [42] described how a logic model based upon the established Implementation Research Logic Model (IRLM) [43] was adapted over time for different projects and contexts within a larger program. The original logic model underwent substantial changes in each project, and the different versions were a helpful tool for documenting similarities and differences between projects. In this way, they supported the identification of key elements of the intervention, effective implementation strategies and contextual challenges. In contrast, the usefulness of our logic model for the adaptation of the new intervention is yet to be seen. Generally, the literature documenting experiences with adapting a logic model to new contexts is limited, leaving an important task for future research.

The additional lessons learnt refer to logic model development in general: First, we chose a rather simple system-based logic model that did not depict relationships between individual domains of the model. Particularly, contrasting with IRLM-based approaches [43], we did not specify which elements of the intervention lead to which outcomes. In our case, we refrained from depicting detailed relationships to facilitate easy communication with policy and practice stakeholders, but also because we found it hard to connect individual program activities to specific outcomes – a challenge that was also encountered by Glasgow et al. [42]. We argue that it may not always be the best solution to create logic models that depict the intervention and its mechanisms in a lot of detail. Still, it remains a task for future research to explore when a more complex logic model and when a simpler logic model represents the most useful approach, and how perspectives on the usefulness of such logic models differ between researchers and policy and practice stakeholders.

Second, logic models can be a useful tool for reflection during the planning and conduct of an evaluation. Specifically, engaging with a logic model at regular intervals, whether in a staged or an iterative manner, enables the evaluation team to check whether they are on track and to uncover potential evaluation blindspots. The early versions of our logic model highlighted the importance of intermediate outcomes – changes to the networking structure and individual gains in expertise – and consequently information on these intermediate outcomes was collected in repeated online surveys. However, we cannot formally examine whether the survey for measuring intermediate outcomes would not have been developed in a similar way without the logic model.

Finally, while municipal stakeholders considered the logic model to be valuable for communication, planning, analysis and quality control, we did not collect data to assess to what extent they really used the logic model for these purposes. In fact, stakeholders still considered the logic model to be complex. Previous research indicates that the complexity of logic models can be a barrier for stakeholders to apply them in practical work [44]. We consider it a drawback of our study that we did not engage policy and practice stakeholders earlier, and that we did not assess for what purposes they would want to use the model. We therefore suggest that the perspectives of all important stakeholders – researchers as well as policy-and-practice stakeholders – are considered at the beginning of the development process, thereby increasing the logic model’s relevance and impact. Established concepts and frameworks can guide this process, among others, the implementation outcomes suggested by Proctor et al. [45], the CICI framework by Pfadenhauer et al. [35] or the IRLM by Smith et al. [43]. Importantly, what researchers consider to be useful does not necessarily match practitioners’ needs; which factors likely contribute to the success of an intervention always has to be informed by practitioners and their understanding of the intervention and its context [2]. Generally, more research is required to understand better how and under what conditions logic models offer practical relevance and concrete benefits to various stakeholders engaged with developing, financing or implementing public health interventions.

## Conclusions

Logic models can be a useful tool when conceptualizing, implementing and evaluating complex interventions in public health. Different approaches to designing logic models exist, and the most suitable approach should be chosen by taking resources and needs related to a given project and the stakeholders involved into account. This study provides a practical example for the development of a logic model in a staged manner, and reflects on the advantages and challenges associated with this approach, as well as the usefulness of the resulting logic model for stakeholders. Still, more such reports on experiences with logic model development processes are needed, to better understand when, and why, to use different approaches and how to implement them. Advancing the development of logic models has the potential to improve the research and practical work on public health interventions, and therefore support the development and implementation of interventions that reach their intended goals.

## Electronic supplementary material

Below is the link to the electronic supplementary material.

Supplementary Material 1

## Data Availability

Protocols of the two workshops with members with the advisory group of Präventionskette Freiham are available on reasonable request from the first author (Stephan Voss, svoss@ibe.med.uni-muenchen.de).

## Abbreviations

CICI framework: Context and Implementation of Complex Interventions framework
IRLM: Implementation Research Logic Model
