# Supplementary material for: Logic models for the evaluation of complex interventions in public health: lessons learnt from a staged development process
Source: BMC Public Health. 2025 May 24;25:1923. doi: 10.1186/s12889-025-23171-8 (PMC12102880; doi:10.1186/s12889-025-23171-8)
Supplement: Supplementary file 1 — Supplementary Material 1 [file 12889_2025_23171_MOESM1_ESM.docx]

***Supplementary Table 1:*** *Changes in the logic model of “Präventionskette Freiham” for each version during development process*

| **Revisions from logic model version 1 to 2** | | | | | |
| --- | --- | --- | --- | --- | --- |
| **Domain** | **Sub-domain** | **Type of revision** | **Specific revision** | **Input for revision** | **Rationale** |
| Intervention | Goal | Wording | Added “promote social participation, reduce health inequalities and promote health” as sub-goals | - Research team reflections | Describe the goals of the intervention in more detail and clarify that promoting health is not the only main goal |
| Intervention | Organization and structure | Content | Deleted “municipal departments” as core element | - Expert interviews with members of the local network and network coordinators - Focus group with advisory group - Research team reflections | Interviews indicated that municipal departments beside steering committee and advisory group are not structural elements of their own |
| Intervention | Organization and structure | Wording | Added “local network” as core organizational element | - Research team reflections | Local network should be depicted as a factor of its own, instead of being subsumed under “Network coordinators” as before |
| Intervention | Program theory/ mechanisms | Content | Added “production network” as core mechanism | - Expert interviews with members of the local network and network coordinators - Focus group with advisory group | In interviews, it was stated as an important distinction from other interventions that *Präventionskette Freiham* works as a “production network” |
| Intervention | Agents of delivery | Content | Added “other” as agent of delivery | - Expert interviews with members of the local network and network coordinators - Focus group with advisory group | In interviews, it became evident that other institutions beyond those already included in the model were relevant as agents of delivery |
| Implementation | Funding | Content | Replaced “projects” by “institutions” as factor | - Expert interviews with members of the local network and network coordinators | General funding situation of institutions was stated as relevant factor in interviews |
| Implementation | Network | Content | Added “continuity” and “public relations” as factors | - Expert interviews with members of the local network and network coordinators | Brought up as a new relevant factor in interviews |
| Context | Infrastructural | Content | Added “existing networks” as infrastructural context factor | - Expert interviews with members of the local network and network coordinators - Focus group with advisory group | Brought up as a new relevant factor in interviews |
| Population |  | Content | Replaced “departments for Health, Social Services and Education” by “municipal departments” | - Expert interviews with members of the local network and network coordinators - Focus group with advisory group - Research team reflections | Clarify that more than the three previously named departments are relevant actors within the intervention |
| **Revisions from logic model version 2 to 3** | | | | | |
| **Domain** | **Sub-domain** | **Type of revision** | **Revision made** | **Source for revision** | **Reasoning** |
| Intervention | Goals | Content | Revised goals as bullet points; added “cultural and educational participation” to “social participation; added “prevent consequences of poverty in children and adolescents” as goal | - Feedback from workshop with advisory group | Stakeholders considered goals as too focused on health and wanted to broaden the scope |
| Intervention | Background | Content | Added “economical and educational disadvantages” to “health-related disadvantages” | - Feedback from workshop with advisory group | Stakeholders considered background as too focused on health and wanted to broaden the scope |
| Intervention | Organization and structure | Content | Added “working groups” as part of the local network | - Feedback from workshop with advisory group | Stakeholders considered the working groups as an elemental part of the local network |
| Intervention | Organization and structure | Content | Added “existing networks” as organizational core element | - Feedback from workshop with advisory group | Networks for families and children that were already present in the Freiham district were considered as a core structural element of the intervention |
| Intervention | Types of delivery | Content | Added “homepage” | - Feedback from workshop with advisory group | During implementation process, it was decided that *Präventionskette Freiham* should get a homepage that could act as a tool for networking |
| Intervention | Agents of delivery | Content | Added “network coordinators” | - Feedback from workshop with advisory group | Stakeholders wanted to highlight the role of the network coordinators as agents of delivery |
| Implementation | Funding | Wording | Added “coordinators” | - Feedback from workshop with advisory group | As securing funding for the network coordinators was a key moment during the implementation process, stakeholders wanted to highlight its significance by dedicating it a separate point, instead of subsuming it under “actors” |
| Implementation | Network | Content | Added “evaluation” | - Feedback from workshop with advisory group | Stakeholders had experienced the evaluation process as a helpful tool for reflecting on the intervention and refining it during implementation |
| Context | Geographic | Content | Added “greenspaces” | - Feedback from workshop with advisory group | Stakeholders considered the amount of greenspaces as a key contextual factor |
| Context | Infrastructural | Wording | Added “health-related, educational and social” as specifications of the existing infrastructure | - Feedback from workshop with advisory group | Stakeholders wanted to go into more detail on which specific kinds of infrastructure they considered as relevant contextual factors |
| Context | Infrastructural | Content | Added “mobility” and “district under construction” | - Feedback from workshop with advisory group | Stakeholders highlighted the importance of traffic and mobility options for the population in the new district as a factor; furthermore, they wanted the specific context of an area under construction to be depicted explicitly in the logic model |
| Population |  | Content | Added “local politics” under “city of Munich” | - Feedback from workshop with advisory group | Stakeholders pointed out that local politicians were an important group that would play a role in the intervention |
| Intermediate outcomes | Structural level | Wording | Added “networking infrastructure” and “accessibility” | - Feedback from workshop with advisory group | Stakeholders wanted to highlight the networking infrastructure as a point of its own instead of subsuming it under support infrastructure; furthermore, it was considered as a relevant intermediate outcome that the accessibility of offers for families and children would be increased |
| Intermediate outcomes | Individual level | Content | Added “increased use of support infrastructure” and “networks” | - Feedback from workshop with advisory group | Stakeholders considered it a relevant intermediate outcome that the refined support infrastructure would also be used more by the target population, and that professionals increased their individual networks |
| Outcomes |  | Structure | Divided “Non health-related outcomes” into outcomes regarding “Education” and “Social” factors; following this, some developmental outcomes were transferred from “Health-related outcomes” to “Education” | - Feedback from workshop with advisory group | Stakeholders wanted to highlight the relevance of outcomes regarding education and social factors |
| Outcomes | Health | Content | Added “well-being” | - Feedback from workshop with advisory group | Identified as an important health-related outcome by stakeholders |
| Outcomes | Education | Content | Deleted “school leavers by graduation” | - Feedback from workshop with advisory group | Stakeholders stated that the degree of school leavers was no indicator that they supposed the intervention to have an effect on |
| Outcomes | Education | Content | Added “successful transitions” | - Feedback from workshop with advisory group | Stakeholders considered this to be an important outcome of the intervention |
| Outcomes | Social | Content | Added “inclusion” and “cultural participation” under “participation” | - Feedback from workshop with advisory group | Stakeholders wanted to add these two aspects of participation as important outcomes |
| **Revisions from logic model version 3 to 4** | | | | | |
| **Domain** | **Sub-domain** | **Type of revision** | **Revision made** | **Source for revision** | **Reasoning** |
| Intervention | Background | Structure | Changed the order of “Background” and “Goals” | - Research team reflections | Improve continuity of this section of the logic model |
| Intervention | Goals | Wording | Reworked the general wording of this sub-section | - Document review | Match with the new official wording of the intervention |
| Intervention | Organization and structure | Content | Deleted “existing networks” from the list | - Research team reflections | At the team-intern workshop, it was decided that these networks were not a genuine part of the intervention, but solely a contextual factor |
| Context | Political | Wording | Changed to “political situation (city and district)” instead of “(national, regional, district)” | - Research team reflections | Focus on the key contextual factors; the national political conditions, while not completely irrelevant, were considered as less important and therefore deleted |
| Context | Epidemiological | Wording | Added “developmental delays” to “spread/prevalence of diseases”, delete regional specification | - Research team reflections | At the team-intern workshop, developmental delays were discussed as a major aspect that should be included separately; the regional specifications were discussed to be not relevant in this sub-section and therefore deleted |
| Intermediate outcomes |  | Structure | Shifted position within logic model; divided into three sub-sections: Structures, Professionals, Target Groups | - Research team reflections - eDelphi on outcome indicators | Intermediate outcomes were moved to a more central position to illustrate their importance for the evaluation of the intervention: likewise, the division into three sub-sections, instead of two, was conducted to depict the intermediate outcomes in more detail |
| Intermediate outcomes | Structures | Wording | Specified networks to be “horizontal and vertical” | - Research team reflections | To illustrate that new networking structures should not only include professionals in the district, but also between professionals in the district and the municipal administration |
| Intermediate outcomes | Professionals | Content | Added “application of acquired competencies and knowledge at work” | - Research team reflections | To reflect that, for the effectiveness of the intervention, it is not sufficient that the professionals gain new competencies or knowledge, but they also have to apply it |
| Intermediate outcomes | Target groups | Content | Added “behaviour changes” | - Research team reflections | To reflect that, for the effectiveness of the intervention, it is not sufficient that the target groups gain new competencies or knowledge, but that these need to result in behaviour changes also |
| Outcomes | Health | Content | Added “nutrition” and “physical activity” | - eDelphi on outcome indicators | Both indicators were considered to be important outcomes by experts in the eDelphi |
| Outcomes | Health | Wording | Changed sub-heading “Prevention” to “Health behaviour” | - eDelphi on outcome indicators | Adding “nutrition” and “physical activity” as outcomes required to change the sub-heading to fit the scope of this section |
| Outcomes | Social | Content | Summarized the bullet points under “participation” to “social and cultural participation” | - Research team reflections | The more general wording was used to include all types of participation under this point, not only the ones mentioned before |
| Outcomes | Social | Content | Added “proportion of children/adolescents affected by poverty” | - eDelphi on outcome indicators | Named as a relevant outcome by experts in the eDelphi |
